# Genetic diversity and population structure analyses in barley (*Hordeum vulgare*) against corn-leaf aphid, *Rhopalosiphum maidis* (Fitch)

**DOI:** 10.3389/fpls.2023.1188627

**Published:** 2023-09-06

**Authors:** Sunny Maanju, Poonam Jasrotia, Surender Singh Yadav, Preeti Sharma, Prem Lal Kashyap, Sudheer Kumar, Manoj Kumar Jat, Gyanendra Pratap Singh

**Affiliations:** ^1^ Division of Crop Protection, Indian Council of Agricultural Research-Indian Institute of Wheat and Barley Research, Karnal, Haryana, India; ^2^ Department of Entomology, Chaudhary Charan Singh Haryana Agricultural University, Hisar, Haryana, India

**Keywords:** aphids, host plant resistance, genetic variation, phylogeny, cereals

## Abstract

Corn-leaf aphid (CLA), *Rhopalosiphum maidis* (Fitch) (Hemiptera: Aphididae) is a serious economic pest of barley worldwide. Breeding for aphid resistance in plants is considered a cost-effective and environmentally safe approach for aphid control, compared to the use of chemical pesticides. One of the challenges in breeding for aphid resistance is the identification of resistant plant genotypes, which can be achieved through the use of molecular markers. In the present study, a set of aphid specific 10 simple-sequence repeats (SSR) markers were used to investigate genetic diversity and population structure analyses in 109 barley genotypes against *R. maidis.* Three statistical methods viz., multivariate hierarchical clustering based on Jaccard’s similarity coefficient, principal coordinate analysis (PCoA) and the Bayesian approach were utilized to classify the 109 barley genotypes. The analyses revealed four subpopulations i.e., SubPop1, SubPop2, SubPop3 and SubPop4 with 19, 46, 20 and 24 genotypes including admixtures, respectively and represented 17.43%, 42.2%, 18.34% and 22.01% genotypes of the total population size, respectively. The studied SSR markers produced 67 polymorphic bands, with an average of 6.7 and ranging from 3 to 12 bands. Heterozygosity (H) was found to be highest in SSR28 (0.64) and lowest in SSR27 (0.89). The observed genetic diversity index varied from 0.10 to 0.34 (with an average of 0.19). Major allele frequency varied from 74.08% to 94.80%. On an average, 87.52% of the 109 barley genotypes shared a common major allele at any locus. Based on the Aphid Infestation Index (AII), only 2 genotypes were found to be resistant against CLA. SubPop2 also had lowest mean aphid population (28.83), widest genetic similarity index (0.60-1.00) and highest genetic similarity coefficient (0.82), which highlighted its potential for inclusion in future CLA resistance breeding programs.

## Introduction

Barley (*Hordeum vulgare* L.) is the fourth most important cereal grain crop after maize, wheat and rice, which is currently cultivated on 52.66 million hectares of land worldwide (Kumar et al., 2022). It is commonly used as staple food in some parts of the world, livestock feed and malt in the brewing industry globally (Nyiraguhirwa et al., 2022). Although, it is a temperate crop, but it can be successfully grown under tropical and subtropical climatic conditions. Out of approximately 4,700 species in the Aphididae family, 183 (3.9%) are crop pests (Hardie, 2017). Amongst them, seven aphid species have been reported to attack barley crop worldwide (Weibull et al., 2003). These aphid species include *Diuraphis noxia* (Mordv.), *Metopolophium dirhodum* (Walk.), *Rhopalosiphum maidis* (Fitch), *Rhopalosiphum padi* (L.), *Schizaphis graminum* (Rond.), *Sipha flava* (Forbes) and *Sitobion avenae* (Fabr.). Corn-leaf aphid (CLA), *R. maidis* (Hemiptera: Aphididae) is the single key aphid species attacking barley in North Western Plains Zone of India (Malik et al., 2013). It is a specialist monocot herbivore and a polyphagous pest which feeds on barley, corn, sorghum, oats, wheat and many more plants from the families gramineae, cyperaceae and typhaceae. Among these host plants barley is most preferred host of CLA (Kaur and Deol, 1999). Aphids have short generation time and parthenogenetic reproduction (Blackman and Eastop, 2000; Ryalls and Harrington, 2017) along with continuous phloem desaping by both adults and nymphs (Bale et al., 2008) and vectoring various viruses like Barley yellow dwarf virus (BYDV) (Rochow, 1979; Straub and Boothroyd, 1980; Irwin and Goodman, 1981), causes yellowing (chlorosis), curling (leaf rolling) and subsequent drying (necrosis) of leaves which ultimately lead to reduction in number and size of earheads (Bhatia and Singh, 1977). The losses in yield reported to be caused by *R. maidis* varied from 17.1% to 100% in barley in India (Murty et al., 1968; Bhatia et al., 1973; Chhillar and Verma, 1982). Aphid infestation on crop plants is currently managed with the use of mainly systemic pesticides, which may pose a threat to the environment, kill non-target beneficial insects (predators, parasitoids and pollinators) and pose risk of aphids developing resistance to the insecticides. In view of the above situation, there is an utmost need to develop alternative control measures for aphids, such as breeding for host plant resistance (HPR). Developing insect-resistant cultivars is an environment friendly, efficient and easy to use method for the farmers (Singh, 2011). Presently, none of the cultivated barley variety in India is resistant to CLA (Porter et al., 2019; Singh et al., 2022). Although, some wheat and barley cultivars have been reported as resistant to one or both of *S. graminis* and *D. noxia* (Mornhinweg et al., 2012; Tolmay et al., 2016; Mornhinweg et al., 2017). Now there is an urgent need to identify underlying resistance mechanisms as well as morphological and biochemical characterization of aphid resistance in barley. A screening of 121 winter barley cultivars for *R. maidis* resistance detected high levels of resistance in seven entries (Hormchong and Wood, 1963). Unfortunately, two of the resistant entries (“Davie” and “Rogers”) from this study were susceptible to *S. graminum.* Another line in this study (PI87181) was susceptible to corn leaf aphid, but was resistant to greenbug. These differential plant responses indicate that different genes control resistance to the two aphid species (Porter et al., 2019). Screening of barley germplasm consisting of about 5000 lines (acquired from National Bureau Plant Genetic Resources, New Delhi) against CLA at Punjab Agricultural University has led to the identification of nine barley genotypes with high level of resistance (Singh et al., 2022). Screening of 474 barley cultivars by Hsu and Robinson (1962; Hsu and Robinson, 1963) from the Canadian Genetic Stock of Barley showed 41 selections with tolerance and antibiosis resistance to *R. padi*. However, none of the 474 cultivars in the collection showed very high levels of aphid resistance, and only 43 selections (9%) were considered sources of resistance for breeding purposes. Moderate to high level of resistance has been observed on screening of barley germplasm for resistance to CLA in India (Verma, 1993; Verma and Nagarajan, 1996; Singh et al., 2006; Singh and Singh, 2009). Since last 40 years, only one resistance source (EB-921) has been utilized in barley breeding program globally (Singh et al., 2022). However, its hybrid derivative, DL-117 is more resistant than EB-921 (Gill and Metcalfe, 1977). *Hordeum* species other than cultivated barley (*H. vulgare* ssp. *vulgare*) have been evaluated as potential sources of resistance to *R. padi*. A diploid species, *H. bogdani* Wilenski, had very high levels of resistance, but genetic incompatibilities between this species and *H. vulgare* make it difficult to transfer the resistance to cultivated barley (Porter et al., 2019). However, the resistance genes from the wild species (*H. vulgare* ssp *spontaneum*, the progenitor of cultivated barley) can be transferred into cultivated varieties through introgression breeding. Due to the difficult and time consuming process for screening aphid resistance in conventional barley breeding programs, gene specific or closely linked markers have also been deployed for indirect selection of phenotype at allele level for molecular marker assisted selection (MAS). The resistance to *R. maidis* is governed by one or two genes with dominant or recessive reaction (Gill and Metcalfe, 1977; Dayani and Bakshi, 1978; Yadav, 2003; Singh et al., 2022). Marker Assisted Selection (MAS) is the best method to select *R. maidis* tolerant genotypes during aphid resistance breeding program and to incorporate resistance in susceptible barley varieties (Malik et al., 2013). The PCR based molecular markers became dominant in evaluation of different traits at DNA level with the availability of SSR based high density maps in barley (Varshney et. al., 2007). Malik et al. (2013) identified RAPD primer OPAC-01 as closely linked marker for *R. maidis* resistance in barley. Two markers KV1/KV2 and SCSSR15864 were effective in identifying genomic region that provides resistance against corn-leaf aphid in barley (Malik et al., 2012). In view of the above background, the genetic diversity and population structure analyses in barley was carried out to identify sources of resistance against corn-leaf aphid, *R. maidis* using microsatellite markers.

## Materials and methods

### Genotype screening

The screening and lab experiments were carried out at ICAR-Indian Institute of Wheat and Barley Research (IIWBR), Karnal (India) Entomology Laboratory and Research Farm during *rabi* season 2021-22. A total of 109 cultivated barley (*H. vulgare*) genotypes acquired from Germplasm Resources Unit (GRU) facility of IIWBR, were screened for determining their resistance response against *R. maidis*. The list of barley genotypes screened during the investigation has been listed in the **Supplementary Table S1**. Each genotype was sown in one meter row with row to row spacing of 25 cm (3 replications; 2 rows per replication) in randomized block design (RBD) following all crop package and practices but without any insecticidal application. Each genotype was tagged with the genotype code and name of the genotype. The genotype screening against aphid infestation in terms of aphid counts per shoot was done three times during the season from five randomly selected plants from each genotype row. The categorization of barley genotypes was done on the basis of peak aphid incidence to increase the selection pressure by following grading system as suggested by Zhu et al. (2005) described in the **Supplementary Table S2**. Based on these gradings, an Aphid Infestation index (AII) was developed as per Kumar et al. (2020) with some modifications. AII was weighted in 3:2:1 for aphids per shoot, leaf chlorosis and leaf rolling respectively, to form an index of 0 to 5 ordinal scale with 0 being immune and 5 being highly susceptible genotype.

### Plant material sampling and DNA extraction

Four to five tender leaf samples from month-old barley seedlings of all 109 barley genotypes were cut using scissors and collected in aluminium packets from each genotype kept in an ice box. Genomic DNA was isolated from 30-day-old seedling following a 3-day long modified Cetyl Trimethyl Ammonium Bromide (CTAB) extraction method given by Saghai-Maroof et al. (1984). To each DNA sample, 3µl of ready-to-use RNase of 10mg/ml concentration was added to degrade the RNA impurities present in the sample. The quality (stock DNA purity: A_260_/A_280_ absorbance ratio) and quantity (stock DNA concentration) in ng/μl from 1 µl sample of stock DNA of each genotype was determined using ScanDrop Spectrophotometer (Analytik Jena) to prepare working DNA solutions. Working solution of each genotype was prepared by reducing the DNA concentration to 50 ng/μl by adding NFW (Nuclease Free Water).

In the current study, a total of 15 simple sequence repeat (SSR) primers from barley genome were initially selected after reviewing the available literature. After preliminary primer runs, ten polymorphic SSR primers (**Table 1**) specific to aphid were shortlisted by eliminating the five monomorphic primers for screening of barley genotypes against *R. maidis* infestation.

**Table 1:** Genetic characteristics of ten polymorphic SSR markers in 109 barley genotypes.

Locus	Primer Sequence (5’-3’)	Repeat motif	Amplicon size range (bp)	Number of alleles	Major Allele Frequency (%)	Gene Diversity	PIC	H
SSR3	F: TAGCCTTTGAGGTCGATGTAGG	(GTT)_5_	150-600	7	89.64%	0.17	0.81	0.83
	R: TGGGTGTCTTTCAGATGAGTTG							
SSR4	F: CAATTACTCGTCGTCCTCCTTC	(TCC)_6_	150-250	3	89.60%	0.17	0.58	0.65
	R: AGCGTTCAGCGTAAGGTAGTTC							
SSR5	F: GCTCGTCTACCTCTGCGATACT	(GGA)_5_	150-300	4	74.08%	0.34	0.69	0.73
	R: TCTGCATCTCAATCAACCAATC							
SSR17	F: TCTTGTGGAGTCTGCTGTTGTT	(TGC)_5_	100-500	7	91.87%	0.14	0.80	0.82
	R: GTAGCTTCAGGTCGCATCACTT							
SSR20	F: GGTTGTTGTCATAGGGGTTGTC	(TCG)_4_	100-500	7	87.68%	0.19	0.80	0.82
	R: TACCAGAACATGGGTTTCAGC							
SSR21	F: AGGTCTGGTGTGAGTGTTGATG	(TGA)_4_	200-500	6	88.83%	0.14	0.79	0.82
	R: CTCCTCATTGTAGTGCGTGTGT							
SSR25	F: TACTTCTCCTCCTCCTCCTCCT	(TATT)_5_	100-600	9	82.87%	0.24	0.79	0.81
	R: GAACTCGCAAAGTGGTTTCTCT							
SSR27	F: CATTTCAGTGTTGGACAAGCAT	(GTGTCA)_4_	100-800	12	87.76%	0.20	0.88	0.89
	R: AGAGAGTTTCGTAGTTGGGCAG							
SSR28	F: CTAAGCATAAGGAGGCAACCAG	(TAAAA)_5_	150-300	3	94.80%	0.10	0.57	0.64
	R: CGGAGTATTGGGAGTGAAATGT							
SSR31	F: CACAAACACACACACACACACA	(GCTCCC)_4_	150-800	9	88.68%	0.19	0.85	0.86
	R: CTGAACAGTAAAGCCTGAAGGG							
**Average**	–	–	–	6.7	87.52%	0.19	0.76	0.79

F, forward primer; R, Reverse primer; T_a_, Annealing temperature; H, Heterozygosity; PIC, Polymorphic Information Content.

The primer stock of 100 pmol/μl concentration was diluted to prepare working primer solution by adding (10 μl) stock primer solution to NFW (90 μl) in the ratio 1:9. The primers were synthesized by Eurofins Genomics India Pvt. Ltd., Bangalore. The primer sequences and repeat motifs of these ten SSR markers are listed in **Table 1**.

### Polymerase chain reaction

A 10µl PCR reaction was undertaken with 5 µl master-mix (GoTaq^®^ Green Master Mix), 0.3µl of forward and reverse primers (Eurofins) each, 1 µl DNA (50 ng/μl) and 3.9 µl of NFW. The amplification reactions were performed on 96 gradient Q-Cycler 96 (Hain Lifescience, United Kingdom). The PCR reactions were run under the following conditions at the annealing temperature corresponding to the primer used: using the following thermal cycling parameters: initial denaturation (94°C for 4 minutes), followed by 35 cycles of denaturation (94°C for 1 minute), annealing at temperatures of 52°C (SSR25 and SSR27), 54°C (SSR3, SSR4, SSR17, SSR20, SSR21, SSR28 and SSR31) and 55°C (SSR5) corresponding to each primer pair for 60 seconds, extension at 72°C for 1 minute, and a final extension step at 72°C for 7 minutes. PCR amplification products were resolved electrophoretically on 3% high-resolution agarose gel stained with ethidium bromide by electrophoresis at 90 V for 1.5 h along with 5μl 100 bp DNA ladder (Promega) for amplicon size comparision; followed by visualization and image capture under UV gel documentation system (Vilber). For each SSR primer pair, amplicons of the same size across different isolates were considered to be the same allele.

### Analysis of population structure and phylogenetic relationships

The electrophoretic bands were scored as 1 (present) or 0 (absent) to form a raw data matrix for further analysis. The genotypic data of number of alleles per marker and base pair size of the alleles was used to calculate the Polymorphic Information Content (PIC) and Heterozygosity (H) using Gene-Calc online tool (Bińkowski and Miks, 2018). Based on Jaccard’s similarity coefficient, the unweighted pair group method with arithmetic averages (UPGMA) clustering method was used to construct a genetic similarity matrix in NTSYS-pc (Numerical Taxonomy and Multivariate Analysis System for personal computer) version 2.0 (Exeter software, NewYork, USA) software package (Rohlf, 2000). PowerMarker version 3.25 (Liu and Muse, 2005), summary statistics included the following: the number of alleles, the major allele frequency and gene diversity. In order to assess the population structure of the 109 barley genotypes, three different statistical methods were adopted and compared. First, a clustering approach based on the Bayesian model was applied to estimate the real number of subpopulations (K) using the admixture model of STRUCTURE software 2.3.4 with correlated allele frequencies (Pritchard et al., 2000; Falush et al., 2003). Three replications in the form of independent runs were performed for each hypothetical number of subpopulations (K) from one to ten applying a burn-in period of 100,000 iterations followed by 100,000 Markov Chain Monte Carlo iterations (MCMC Reps) to obtain a precise parameter estimate. Output of analysis was collected using the STRUCTURE harvester (Earl and VonHoldt, 2012). The most probable expected number of subpopulations (indicated by best/highest K value) was determined using an *ad hoc* statistic by Evanno’s ΔK (Evanno et al., 2005). Each genotype was assigned to one of the subpopulations based on a membership probability coefficient ≥ 0.70. The admixtures were grouped in a separate group of “Mixed” subpopulation; however, their exact subpopulation group was identified by performing the K-means clustering in PAST 4.08 software. These genotypes were sorted by cluster membership coefficient (Q) which represents the probability of an individual belonging, partially or fully to one or more subpopulations under investigation and clubs them together in ordinal manner. Principal coordinate analysis (PCoA) was then carried out using PAST software version 4.08 to visualize the genetic stratification within the barley germplasm collection based on genetic correlations among individuals (Hammer et al., 2001). Thirdly, the phylogenetic relationship between the 109 barley genotypes was estimated using OriginPro^®^ 2022 (OriginLab Corporation, USA) software with the multivariate hierarchical cluster analysis based on marker data. The genetic distance matrix based on Jaccard’s similarity coefficient was applied to construct a circular phylogenetic tree/dendrogram. In order to summarize the major patterns of variation within the multi-locus dataset, an analysis of molecular variance (AMOVA) using GenAlEx (Genetic Analysis in Excel) 6.502 (Peakall and Smouse, 2006) was also performed.

### Statistical analysis

The phenotypic values of number of aphids infesting the 109 barley genotypes were grouped based on the subpopulations obtained in the STRUCTURE cluster analysis. The variations among the whole population and between subpopulations was compared using descriptive statistics boxplot with median, range, interquartile range and subpopulation size in SPSS software (SPSS Statistics 25.0, IBM). One-way analysis of variance (ANOVA) (SPSS Statistics 25.0, IBM) was conducted to compare the means number of aphids infesting genotypes of various subpopulations at the *p* < 0.05 level of significance. Further, to check for individual differences among subpopulations *post-hoc* comparisons were made using Dunnett’s T3 test. All the genotypes were classified into various resistance responses based on Aphid Infestation Index (AII) using a pie-chart (Excel 2019, Microsoft Company). At last, the data of aphids per shoot, AII based resistance responses and the subpopulation categorization by STRUCTURE was integrated in a Wind- Rose Chart (Excel 2019, Microsoft Company) for identification of the desired aphid resistant genotypes and breeding material.

## Results

### Population stratification

**Figure 1:**
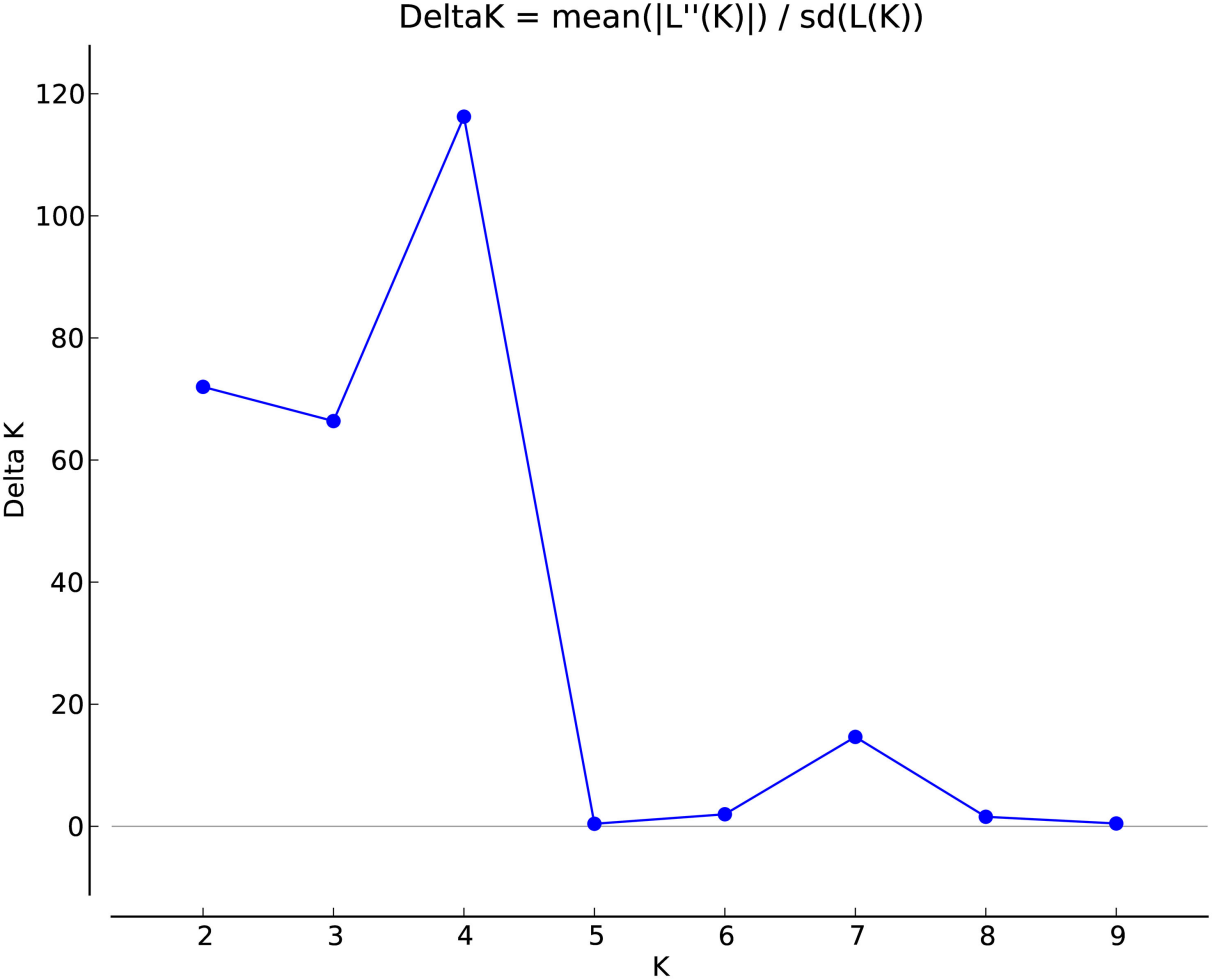
Estimation of the genetically most probable number of *H. vulgare* subpopulations based on Evanno’s Delta K (ΔK) method.

The marker data was analysed by STRUCTURE Harvester software following the ΔK method for K = 1 to K = 10. The data revealed the maximum value of delta K occurred at K=4 indicating that the 109 barley evaluated genotypes can be divided into four hypothetical subpopulations (**Figure 1**).

**Figure 2:**
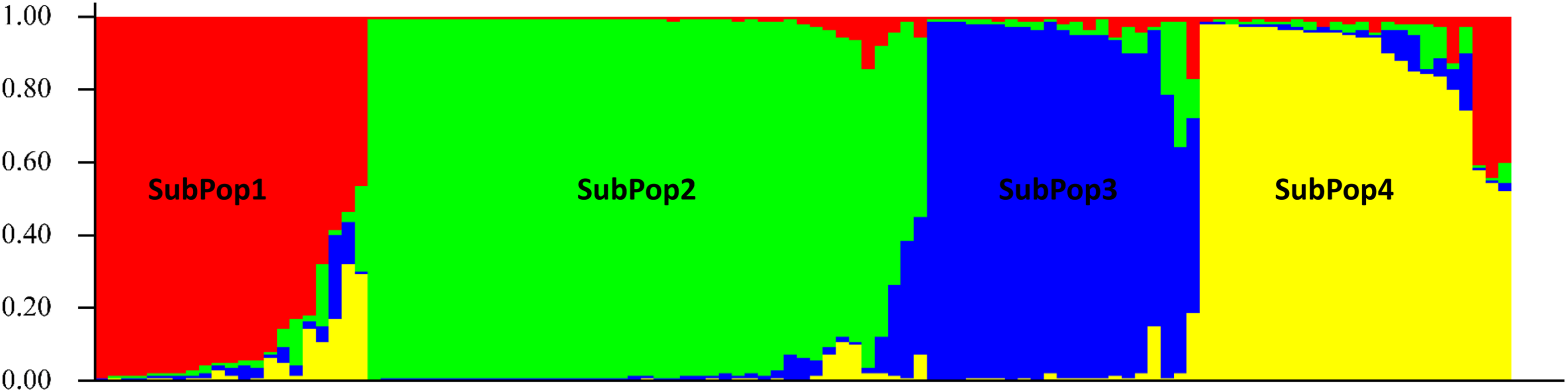
Bar plots for 109 barley genotypes generated by STRUCTURE using the admixture model and sorted by Q at K=4, with independent allele frequency based on binary data of 10 SSR loci. The groups (subpopulations) are represented by different colors. Each bar represents one barley genotype (identified by genotype code) and is partitioned into segments indicating its genetic composition similarity with other genotype groups.

The 109 barley genotypes were grouped into four clusters (as K = 4) and shown in four different colours *viz*. SubPop1 (Red), SubPop2 (Green), SubPop3 (Blue) and SubPop4 (Yellow) with 18, 41, 19 and 20 genotypes, respectively. These genotypes are sorted by cluster membership coefficient (Q) (**Figure 2**). Eleven genotypes showed mixed allelic patterns with a probability of more than 30% that could not be assigned to any of the subpopulations by the STRUCTURE software and hence designated as Mixed subpopulation. Therefore, K-means clustering of genotypes using PAST software identified 11 mixed genotypes as an admixture of one of the subpopulations. SubPop1 had only one admixture i.e., genotype code: 17. SubPop2 had the most admixtures, with 5 admixtures with genotype codes: 23, 25, 56, 57, and 60. Similarly, SubPop3 and SubPop4 have one genotype (genotype code: 49) and four genotypes (genotype codes: 13, 21, 22, 26), respectively. K-means cluster analysis also revealed that the genotypes 25 (Mixed), 57 (Mixed), 28 (SubPop1) and 50 (SubPop2) belonged to their respective subpopulations in spite of being classified in SubPop2, SubPop3, Mixed and Mixed subpopulations by STRUCTURE, respectively. Overall, the four subpopulations (SubPop1, SubPop2, SubPop3 and SubPop4) including their admixture genotypes represented 17.43%, 42.2%, 18.34% and 22.01% genotypes of the total population size, respectively.

### Aphid resistance diversity pattern

**Figure 3:**
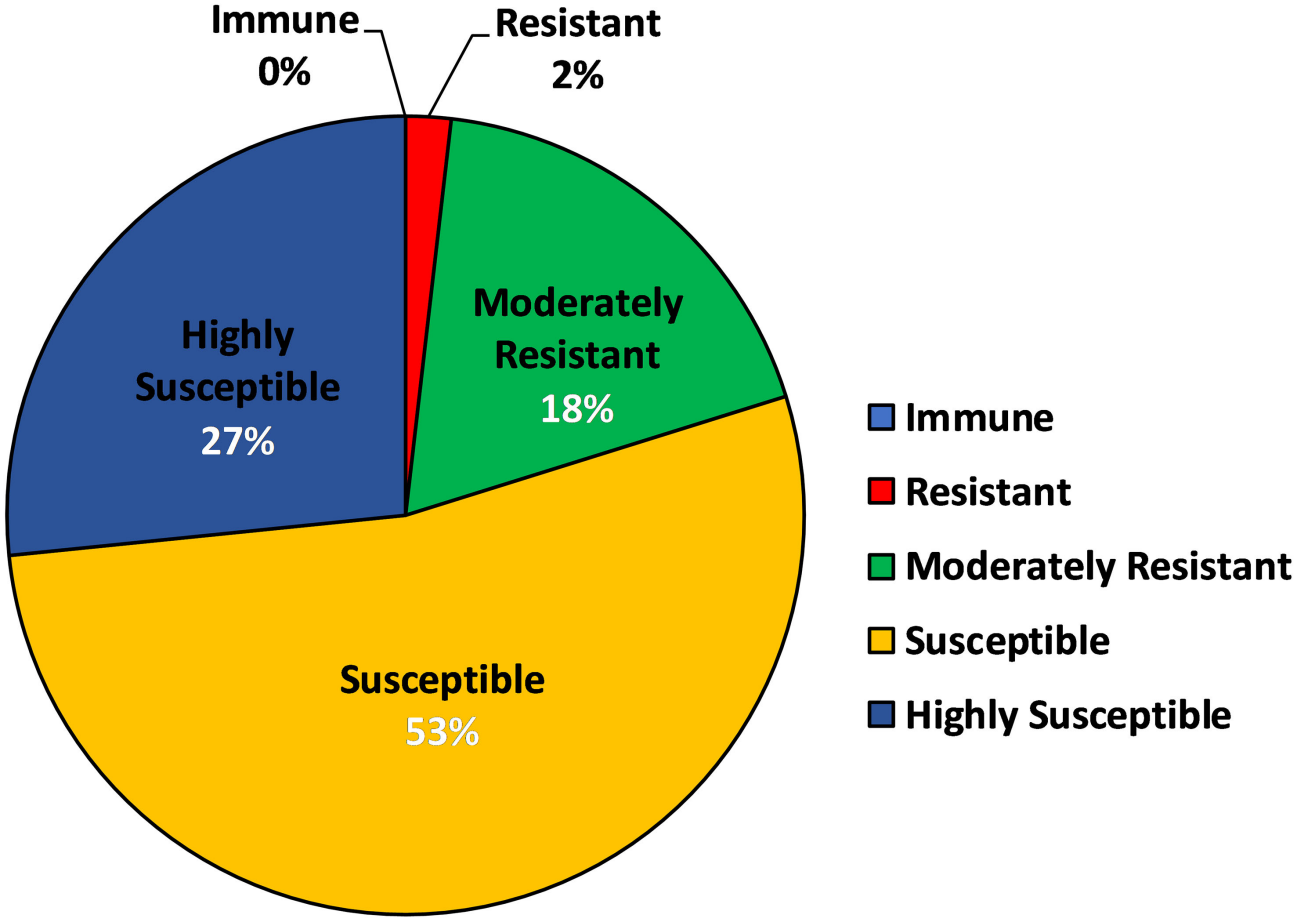
Pie Chart depicting characterization of barley genotypes into categories of resistance based on AII (Aphid Infestation Index).

In order to determine the aphid preference to the various barley genotypes used in the present investigation, an Aphid Infestation Index (AII) was devised on weightage basis, of which highest was given to the number of aphids infesting per shoot, followed by the sequence of symptoms of aphid damage like leaf chlorosis and leaf rolling. Based on the AII, all the genotypes were characterized into 5 categories of resistance response *viz*. immune, resistant, moderately resistant, susceptible and highly susceptible (**Supplementary Table S3**) with 0%, 1.83%, 18.34%, 53.21% and 26.60% genotypes (**Figure 3**), respectively. There was no immune genotype identified in the current study, however, a total of 22 genotypes, which represented 20% of the total barley genotypes were either found to be resistant or moderately resistant (**Figure 3**). RD 2849 (genotype code 86) and DWRUB 52 (genotype code 23) were the only two genotypes which emerged as resistant to corn-leaf aphid, *R. maidis* (**Supplementary Table S3**).

**Figure 4:**
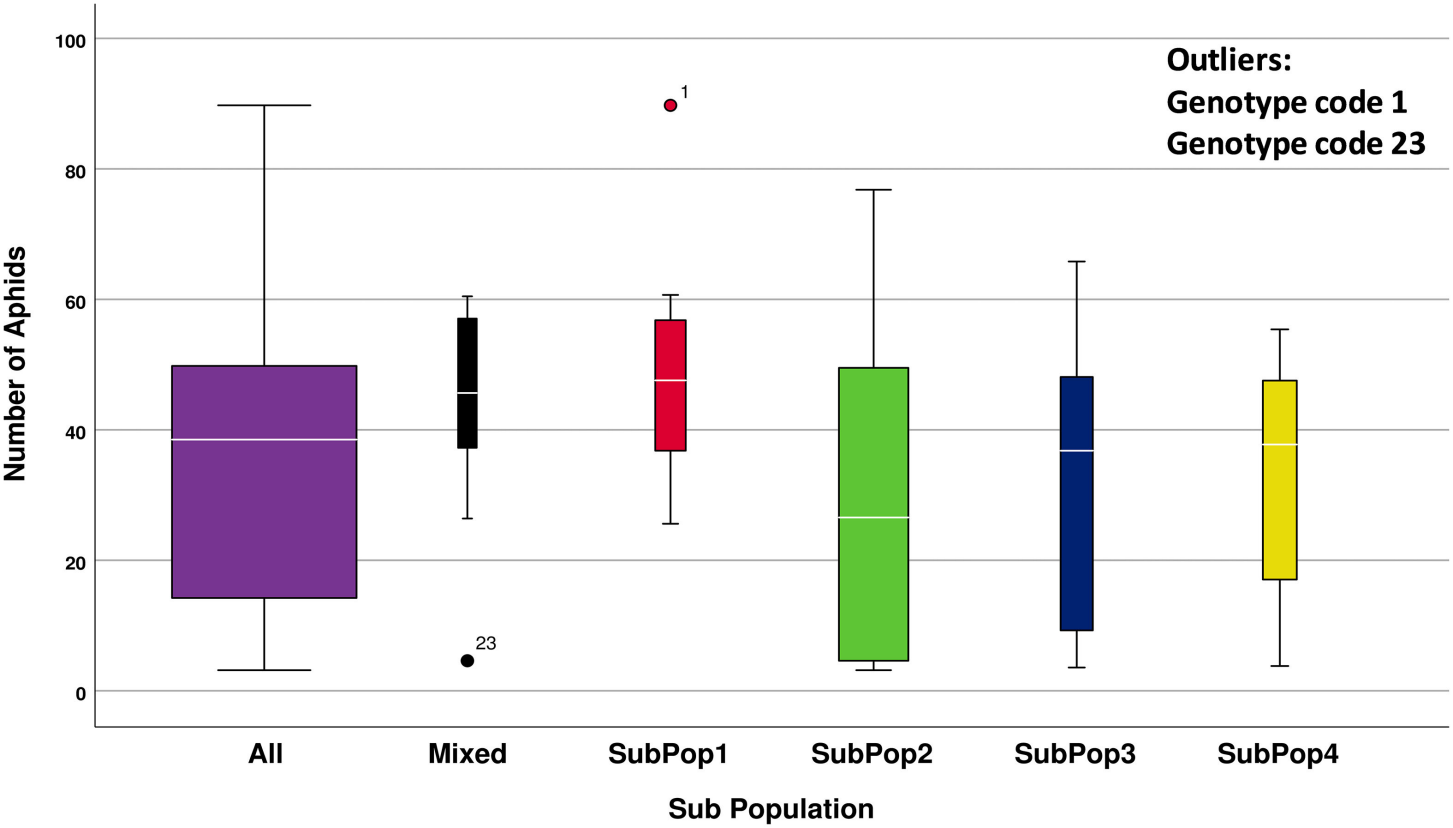
Descriptive statistics of number of *R. maidis* aphids infecting all 109 barley genotypes when divided into 4 subpopulations based on cluster analysis.

Descriptive statistics using box plot diagram using number of aphids infesting per shoot and subpopulation categories have been presented in the **Figure 4**. The number of genotypes included in that subpopulation is indicated by the width of the box. ANOVA results revealed that the mean number of aphids infesting genotypes varied significantly among the different subpopulations (*F*_4,104 =_ 3.757, *p* < 0.05). Since the Levene’s Statistic is significant, the equal variance was not assumed. To check for individual differences between subpopulations *post-hoc* comparisons were assessed using Dunnett’s T3 test. The test indicated that the mean number of aphids infesting SubPop1 (M = 48.31, SD = 14.40) was significantly higher from the mean number of aphids infesting SubPop2 (M = 28.83, SD = 22.97 and SubPop4 (M = 32.41, SD = 17.25). The mean differences were significant at the 0.05 level of significance. However, no significant differences were detected between other subpopulation combinations. Since, SubPop2 (Green) had the highest number of genotypes (46 including admixtures) in it along with the least mean number of aphids (28.83) infesting its genotypes, it emerged as a promising group for search of aphid resistance. Alfa 93 (genotype code 1) being a susceptible check harboured the highest number of aphids and hence classified as an outlier of SubPop1. Contrarily, DWRUB 52 (genotype code 23) being an admixture of SubPop2 have low infestation of aphids and therefore, have been classified as an outlier of Mixed subpopulation.

**Figure 5:**
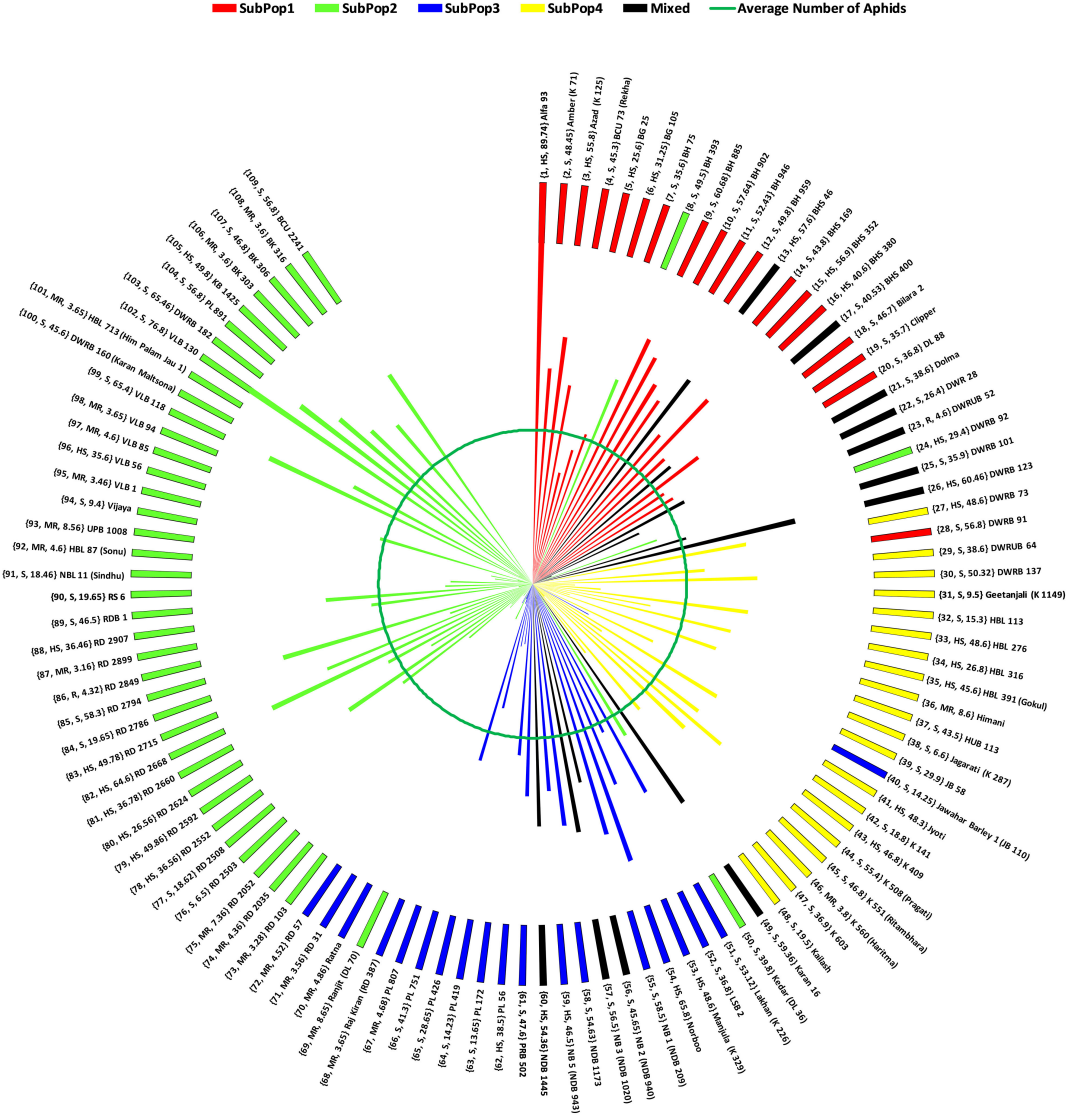
Wind-Rose Chart based on mean number of aphids infesting 109 barley genotypes depicting resistance reaction (AII) and subpopulations (STRUCTURE). Each line representing a genotype has been coloured according to the colour of subpopulation to which the genotype belongs as revealed by STRUCTURE. The inner green circle represents the average number of aphids on all the genotypes (34.52). Each genotype has been labelled in the order: (Genotype Code, Resistance reaction based on AII, Mean number of aphids infesting the genotype) Name of the genotype (alias if any).

Overall, **Figure 5** combines the information on mean number of aphids infesting per shoot, resistance reactions of genotypes based on AII and the subpopulations divided by STRUCTURE cluster analysis, in the form of a wind-rose chart. On comparison, the 63.63% (14 in total) of the genotypes belonging to either resistant or moderately resistant category were found to be from SubPop2 (Green). The two resistant genotypes, identified based on AII, RD 2849 and DWRUB 52 (admixture) also belonged to SubPop2.

### Analysis of genetic diversity

All of the ten SSR markers used in the study produced 67 polymorphic bands, with an average of 6.7 and ranging from 3 to 12 bands. For the SSR loci, polymorphism information content (PIC) varied from 0.57 (SSR28) to 0.88 (SSR27) with an average of 0.76 (**Table 1**). While, heterozygosity (H) indicated the average frequency of heterozygous individual was found to be highest in SSR28 (0.64) and lowest in SSR27 (0.89). The observed genetic diversity index varied from 0.10 to 0.34 (with an average of 0.19). Major allele frequency varied from 74.08% to 94.80%. On average, 87.52% of the 109 barley genotypes shared a common major allele at any locus. The number of bands observed in subpopulations ranged from 26 in SubPop2 to 49 in SubPop1, which all had a frequency of more than 5%. The study found 12 unique alleles detected in 60 barley genotypes spread across each and every subpopulation.

To refine the genetic relationship between the barley genotypes, the genetic similarity within the population was calculated based on Jaccard’s coefficient. The average genetic similarity between all 109 genotypes was estimated to be 0.81 and ranged from 0.60 to 1.00, indicating that the barley genotypes had low genetic variability (data of the genetic similarity matrix not shown). The pair-wise genetic similarity of the genotypes was analysed within each subpopulation, with SubPop2 (Green) having the broadest range of 0.60-1.00 and the highest genetic similarity coefficient of 0.82.

**Figure 6:**
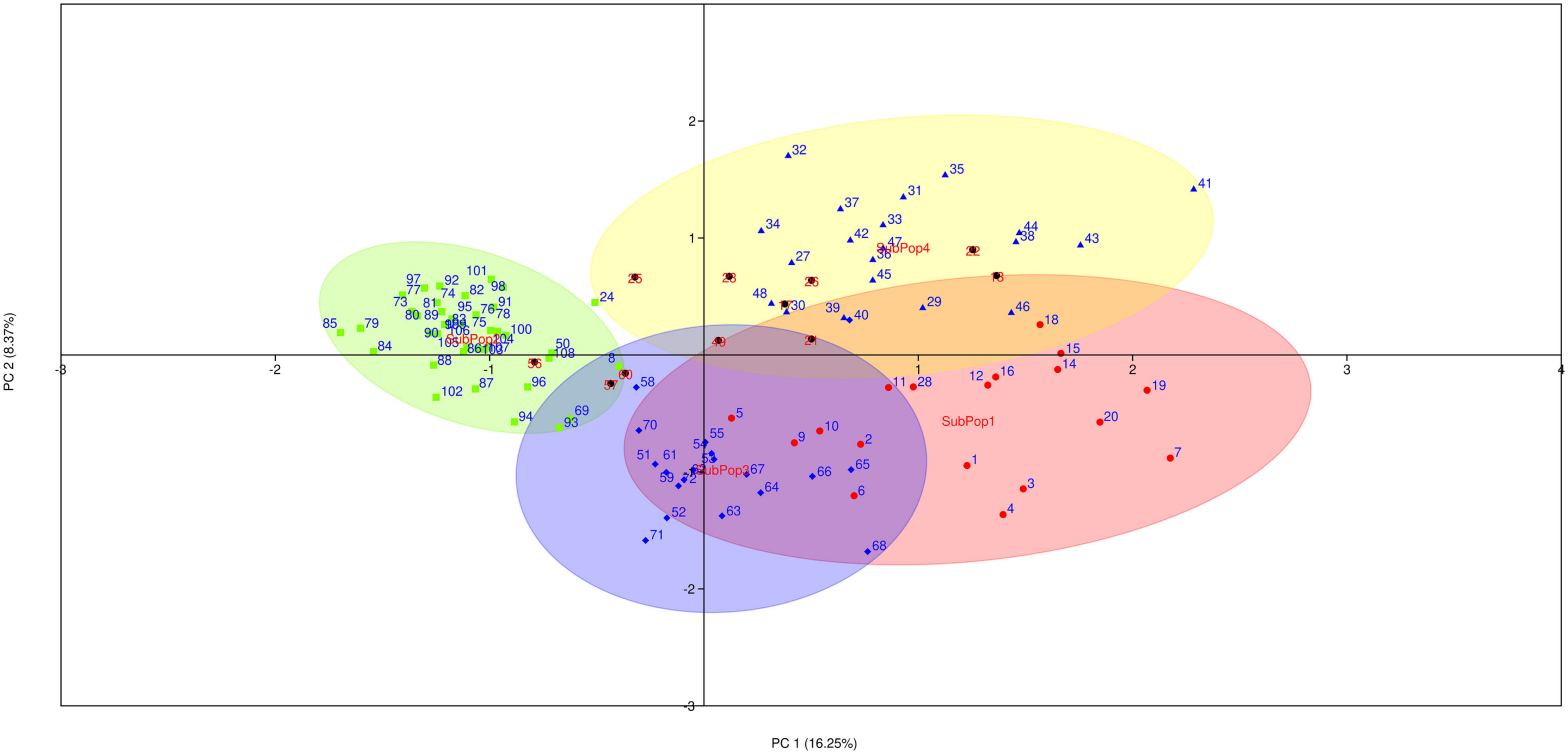
Principal coordinates analysis (PCoA) on 109 barley genotypes based on 10 SSR markers. Different colours indicated different subpopulations (SubPop1-SubPop4) in the population as revealed by STRUCTURE software. The numbers here indicates the genotypes codes.

Principal coordinate analysis (PCoA) was used as an alternative method of analyzing and visualizing the population structure. The first three principal coordinates explained 32.20% of the genotypic variance (PC1: 16.25%, PC2: 8.37%, PC3: 7.58%), and also discriminated the genotypes of SubPop1, SubPop2, SubPop3 and SubPop4 with 95% confidence ellipses, confirming the STRUCTURE analysis results (**Figure 6**). These subpopulations have been coloured in accordance with the colours generated by STRUCTURE software for easy comparison. The cluster of SubPop1 (Red) overlapping with SubPop3 (Blue) and SubPop4 (Yellow) showed their interaction with each other. The compactness of SubPop2 (Green) cluster revealed the high genetic similarity in the subpopulation.

**Figure 7:**
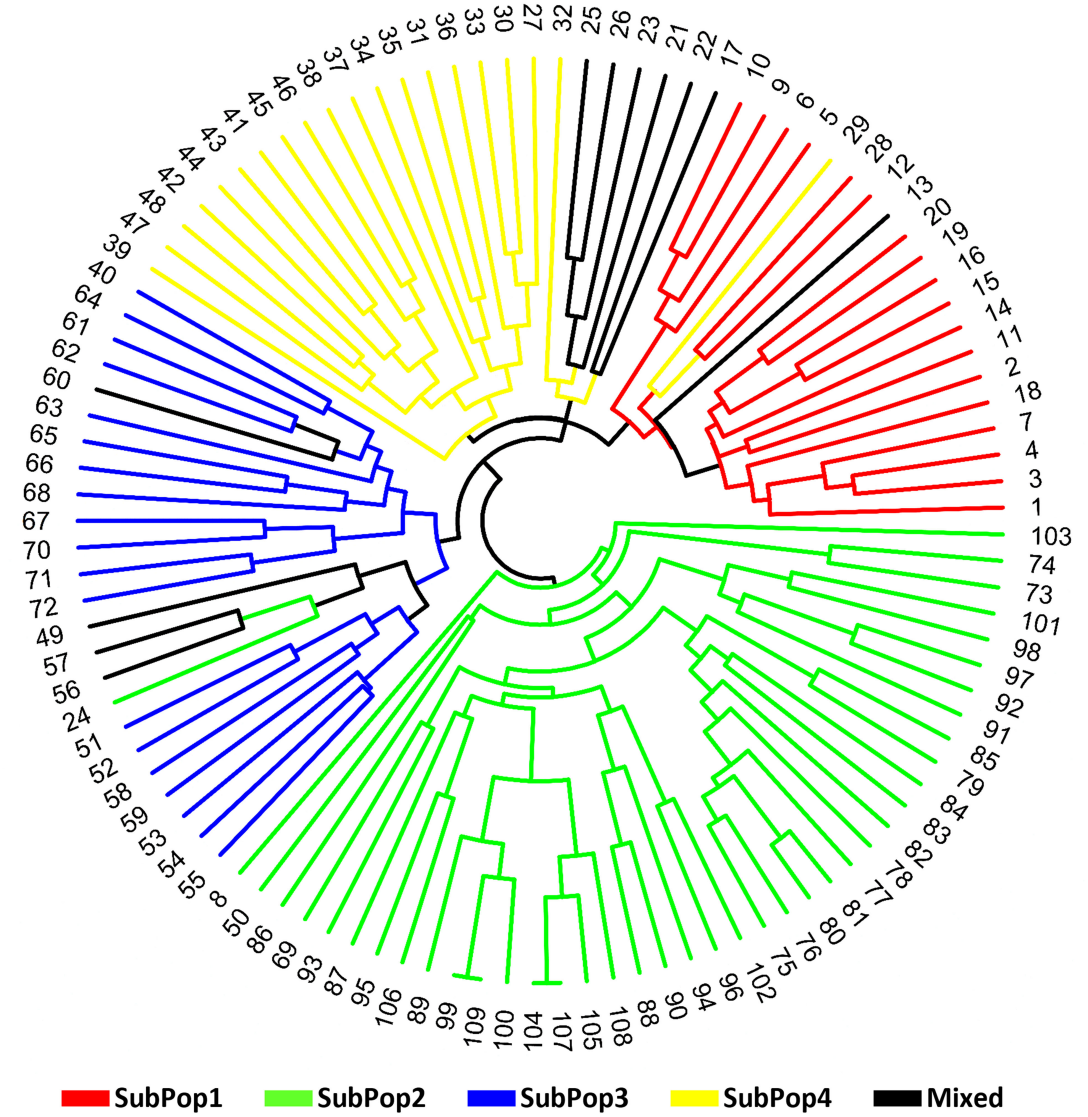
Circular dendogram of 109 barley genotypes constructed from molecular data using OriginPro 2022 software.

Finally, the circular dendrogram generated through multivariate hierarchical cluster analysis using group average method and Jaccard’s similarity coefficient, also grouped all 109 genotypes into four major clusters similar to the four subpopulations (**Figure 7**) identified in STRUCTURE cluster analysis and PCoA. The dendrogram have been coloured as per the colours revealed by the STRUCTURE software for easy comparison.

**Figure 8:**
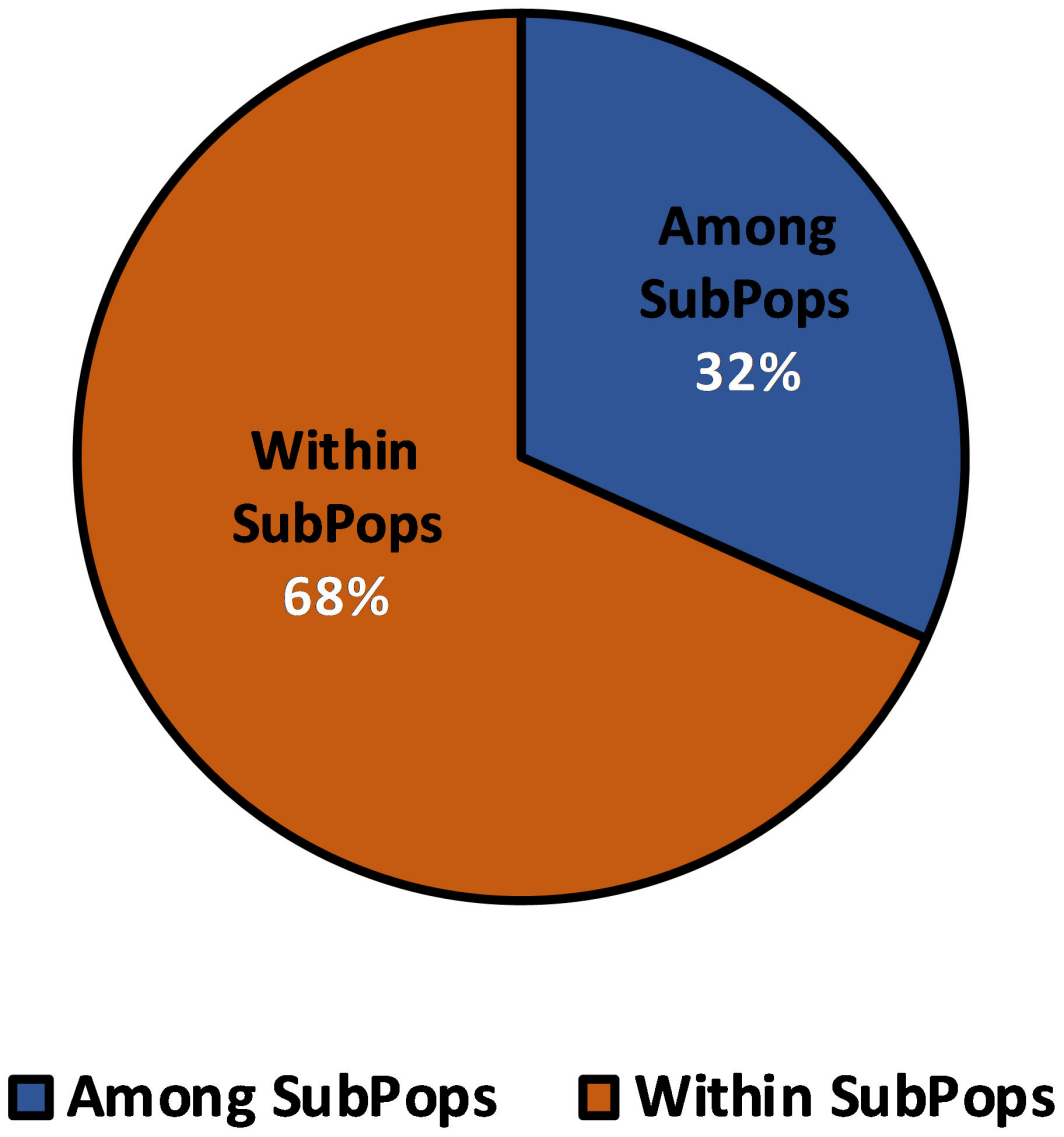
Analysis of Molecular Variance (AMOVA) of 109 barley genotypes based on population obtained by the model-based approach.

The four subpopulations obtained from structural analysis were subsequently exposed to AMOVA in order to determine the variation across and within populations. A 32% variance was observed among subpopulations, while it was 68% within subpopulations (**Figure 8**). This points to a significant gene flow and intra-population differentiation. These subpopulations had some interaction with each other resulting in some variation among them.

## Discussion

Molecular markers are useful tools for characterizing germplasm collections for genetic diversity. They can be used to analyze the genetic variation within and among populations, and to identify the specific genes or regions of the genome that are associated with resistance to biotic stress. This information can be used to identify and select plant varieties with desirable traits for use in breeding programs (Savadi et al., 2021). In addition, population structure and phylogenetic analysis using molecular markers can provide insights into the genetic and evolutionary relationships among different germplasm collections. It can help in identifying groups of genotypes that are genetically distinct with desirable stress tolerance traits and can be used in breeding programs to broaden the genetic base of breeding programs (Sánchez-González et al., 2020). Barley being a self-pollinating crop, examination of genetic diversity becomes important to generate diverse breeding material for insect and disease resistance. Since, there are no reports of studies utilising molecular markers to analyse genetic diversity and population structure in Indian barley genotypes, the present study investigated 109 genotypes of barley for their genetic diversity and population structure analysis using the 10 microsatellite markers and their association with resistance responses against CLA, *R. maidis*. The three statistical methods were used in this study and these gave consistent results for the genetic diversity and population structure. Based on these various clustering analysis, all the 109 genotypes were divided into four subpopulations. Despite, SubPop2 (Green) showing high genetic similarity coefficient (0.82), it clustered genotypes further apart and was found to be the most diverse subpopulation. The maximum genetic similarity coefficient of SubPop2 (0.82) advocates for the maximum similarity among most of the subpopulation genotypes which is a prerequisite for any breeding program, however, a few exceptional genotypes shared striking genetic differences with other SubPop2 genotypes resulting in widening of extreme values of GS coefficient superficially. PCoA also confirmed high degree of similarity in SubPop2 with highest number of genotypes among all the subpopulations clustering together. It also had a significantly low average aphid infestation (28.83) with maximum percentage (63.63%) of resistant genotypes which highlights its potential to be used for hybridization programs in order to develop resistance cultivars against this major pest. Previous study conducted by Chaabane et al. (2009) characterized and differentiated six Tunisian barley varieties (Faïz, Manel, Martin, Rihane, Roho, and Tej) as well as six landraces from different growing regions in Tunisia into two groups using UPGMA cluster analysis of the similarity data. While, Dido et al. (2020) investigated phenotypic diversity and population structure of Ethiopian barley (*Hordeum vulgare* L.) of 585 landrace collections along with 10 cultivars for their phenotypic diversity and population structure in relation to 22 agronomic traits, three major diseases and barley shoot fly resistance-related traits. The study categorized the germplasm into eight clusters based on Euclidean dissimilarity index using the linkage method. Another study by Ferreira et al. (2016) reported the genetic diversity of 64 barley accessions composed of wild and domesticated barley representing genotypes from six countries using 34 microsatellite markers and the UPGMA cluster analysis produced three main clusters. Maniruzzaman et al. (2014) conducted polymorphism study in ten barley (*Hordeum vulgare*) genotypes using eight microsatellite (SSR) markers and divided the whole population into four distinct clusters. Further, Blori-Moghadam et al. (2011) assessed the genetic diversity in seven barley cultivars using 10 microsatellite markers and clustered them separately in 7 groups. While in another study by Nandha and Singh (2014), 27 wild barley and 20 cultivated barley genotypes were grouped into two major clusters of similar genotypes based on the genetic similarity data of both genomic and EST-SSRs, created through UPGMA cluster analysis.

Using SSR marker profiling to identify genetic diversity in barley genotypes, the average number of alleles per locus (6.70) compares to Maniruzzaman et al. (2014), who reported a total of 47 alleles detected by 8 SSR markers among 10 barley genotypes, with an average of 7.8 alleles/locus.Further, Nandha and Singh (2014), found 575 alleles from 47 barley genotypes (20 wild and 27 cultivated) with 6.56 and 5.41 alleles per locus using 48 gSSRs highly polymorphic markers. While, they reported 3.56 alleles per locus in wild and 3.31 alleles per locus in cultivated barley germplasm were generated by 16 EST-based SSR markers. A wide variety of alleles ranging from 2.81 alleles/locus, 8 alleles/locus and 9.28 alleles/locus have been detected by Chaabane et al. (2009); Ferreira et al. (2016) and Blori-Moghadam et al. (2011), respectively. In the present report, PIC values varied from 0.57 (SSR28) to 0.88 (SSR27) with an average of 0.76 which indicates that the ten markers used were highly informative. This accords with the previous values given by Nandha and Singh (2014), who reported PIC values ranging from 0.498 to 0.875 with an average of 0.760 in case of wild genotypes, while 0.180-0.880 PIC values averaging to 0.693 were found in case of cultivated genotypes. PIC values in a study carried out by Blori-Moghadam et al. (2011) were quite high and ranged from 0.80 to 0.88 with an average of 0.84 for the 10 SSR markers used in the study. Similarly, PIC values by Maniruzzaman et al. (2014) also averaged to 0.83 (0.73-0.87 range). Chaabane et al. (2009) and Ferreira et al. (2016), however, reported a range of 0.28-0.60 (with an average of 0.50) and 0.07 to 0.86 (with an average of 0.57), respectively, which are markedly lower than the results in the current study. Lower PIC values often denote more closely related varieties of the germplasm, while on the other hand; higher PIC values can be linked to the use of more informative markers. The findings of this study imply that these SSR markers can prove to be valuable resources for further genetic analysis of barley germplasm.

Identification of unique alleles can have great significance for resistance-breeding purposes. In current study, 12 unique alleles (17.9% of total 67 alleles present) spread across 60 genotypes were identified by 10 SSR markers. Three SSR markers (SSR3, SSR20 and SSR 21) amplified one unique allele, while, one marker each (SSR17, SSR25 and SSR 27) amplified two, three and four alleles, respectively from a total of 60 barley genotypes. Moreover, germplasm with a higher number of unique alleles represents a potential bank of novel alleles for use in a resistance breeding programs. SSR markers have previously identified 36 unique alleles (12.8% of total 280 alleles found) by Ferreira et al. (2016), while, Nandha and Singh (2014) have similarly detected 12 unique alleles in both cultivated and wild barley genotypes which were just 2.08% of total 575 alleles detected. In the present study, genetic diversity ranged from 0.10 and 0.34, averaging 0.19, with a genetic similarity (GS) coefficient of 0.81, reflecting a high level of genetic similarity and low diversity. This GS is comparable to Maniruzzaman et al. (2014) and Chaabane et al. (2009), where an average GS of 0.76 (ranging from 0.74 to 0.83) and 0.62 (ranging from 0.39 to 0.90) were reported. However, Nandha and Singh (2014) detected GS of just 0.28 ranging from 0.04-0.80, indicating genetically diverse genotypes.

Based on the AII, two (1.83%), 20 (18.34%), 58 (53.21%) and 29 (26.60%) of the 109 barley genotypes were characterized as resistance, moderately resistance, susceptible and highly susceptible genotypes to *R. maidis*, respectively. There was no immune genotype identified in the present study. The present findings are in line with that of Hsu and Robinson (1962; Hsu and Robinson, 1963), Chhilar et al. (1985); Singh et al. (2006); Kumar et al. (2020) and Kumar et al. (2022a), who reported that out of all the barley cultivars screened (some but not all of the genotypes were same as used in the current study) against *R. maidis*, none of them was found immune to the pest. Very few sources of resistance are available against corn leaf aphid in India (Singh and Singh, 2009) and so far, no cultivars have been released in India for this trait. However, recently two resistant genotypes BCLA3 and BCLA11-6 have been registered as genetic stocks for novel source of corn leaf aphid resistance at NBPGR, New Delhi (Malik et al., 2022). During peak infestation, highest aphid per shoot incidence was observed in the genotype, Alfa 93 (89.74 aphids/shoot) making it the most susceptible barley genotype. This genotype has also been used by Verma et al. (2011) and Singh and Singh (2009) in their respective screening trials as a susceptible check. The least aphid population was recorded on RD 2899 (3.16 aphids/shoot), which showed contrasting results as compared to Kumar et al. (2020), may be due to the differences in the geographical and/or agronomic conditions under which the genotypes were grown. However, RD 2899 was categorized as moderately resistant genotype according to the AII, in spite of having least aphid infestation. In the present study, 20% genotypes showed some or the other resistance response with RD 2849 and DWRUB 52 found to be the two most *R. maidis* resistant genotypes, both belonging to SubPop2 (Green). The maximum (63.63%) of the resistant genotypes also were from SubPop2. SubPop2 is the most preferred group for research into aphid resistance since it contains the most genotypes (46, including admixtures) and the least mean number of aphids (28.83) infesting its genotypes. The results of AMOVA agreed with results obtained using the phylogenetic tree-based similarity coefficient distribution as well as the structure analysis, with all of these aforementioned approaches confirming the presence of both large genetic similarity and a moderate level of population structure. This represents a critical step for carrying out any future association mapping (AM) analysis.

## Conclusion

Generally, selection of genotypes should be made carefully for any breeding program seeking to create resistance. Cluster analysis from the agro-morphological features grouped all the germplasm into four subpopulations. Significant (*p*<0.05) differences were found among subpopulations, which confirms the presence of diversification among barley genotypes. From the findings of the cluster analysis, the presence of both large genetic similarity and a moderate level of population structure was observed, suggesting that these could be used in resistance breeding programs. As such, the germplasm, as well as the highly polymorphic SSR markers identified in this study, have the potential to facilitate *R. maidis* breeding program for resistance. The findings of this study provide a solid foundation for further work in the effort to fill the gap of resistance sources against corn-leaf aphid in barley.

## Data availability statement

The raw data supporting the conclusions of this article will be made available by the authors, without undue reservation.

## Author contributions

SM, PJ suggested the hypothesis and led the whole study together with PK, SY and MJ. PS, SK and GS assisted in the writing and reviewing of the manuscript. All authors contributed to the article and approved the submitted version.
