# Supplementary material for: Genetic diversity and population structure analyses in barley (Hordeum vulgare) against corn-leaf aphid, Rhopalosiphum maidis (Fitch)
Source: Front Plant Sci. 2023 Sep 6;14:1188627. doi: 10.3389/fpls.2023.1188627 (PMC10510198; doi:10.3389/fpls.2023.1188627)
Supplement: Supplementary file 1 [file Table_1.doc]

**LIST OF SUPPLEMENTARY TABLES**

**Table S1:** List of barley (*Hordeum vulgare*) genotypes under investigation.

| **Genotype code** | **Genotype name** | **Parentage** | **Spike type** | **Year of Release** | **Developed at** | **Geographic Region** |
| --- | --- | --- | --- | --- | --- | --- |
|  | Alfa 93 | AURORA/ QUEEN // BEKA (Introduction) | 2 | 1995 | Karnal | NWPZ |
|  | Amber (K 71) | K12/CN294 | 6 | 1978 | Kanpur | NEPZ |
|  | Azad (K 125) | K12/K19 | 6 | 1982 | Kanpur | NEPZ |
|  | BCU 73 (Rekha) | WUM143 (YAGAN) | 2 | 1997 | Karnal | NWPZ |
|  | BG 25 | C138/CN170 | 6 | 1976 | Hisar | NWPZ |
|  | BG 105 | C141/ MONTLESSO | 6 | 1976 | Hisar | NWPZ |
|  | BH 75 | RD150/AHOR 31/68 | 6 | 1985 | Hisar | NWPZ |
|  | BH 393 | California Mariout/ Ratnal | 6 | 2002 | Hisar | NWPZ |
|  | BH 885 | BH563 ( φSterile) / PL419 | 2 | 2012 | Hisar | NWPZ |
|  | BH 902 | BH495/ RD2552 | 6 | 2010 | Hisar | NWPZ |
|  | BH 946 | BHMS22A/ BH549// RD2552 | 6 | 2014 | Hisar | NWPZ |
|  | BH 959 | BH393/BH331 | 6 | 2015 | Hisar | CZ |
|  | BHS 46 | BHS37-37 / BHS 14-88// KAILASH | 6 | 1984 | Shimla | NHZ |
|  | BHS 169 | KAILASH/ BRIGGS | 6 | 1988 | Shimla | NHZ |
|  | BHS 352 | HBL240/BHS504 //VLB129 | 6 | 2003 | Shimla | NHZ |
|  | BHS 380 | VOILET/MJA/7/ ABN-B6/BA/ GAL// FZA-B /5 /DG/OC-B/ PT-B AR /3/RA-B/BA /3/4/TRYIGAL... | 6 | 2010 | Shimla | NHZ |
|  | BHS 400 | 34^th^ IBON-9009 | 6 | 2014 | Shimla | NHZ |
|  | Bilara 2 | RS17/C251 | 6 | 1980 | Durgapura | NWPZ |
|  | Clipper | Introduction from Australia | 2 | 1969 | New Delhi | NWPZ |
|  | DL 88 | BG1/MEX5-13 | 6 | 1998 | New Delhi | NWPZ |
|  | Dolma | Selection from USA-115 (Introduction) | 6 | 1982 | Bajaura | NHZ |
|  | DWR 28 | BCU73/PL172 | 2 | 2002 | Karnal | NWPZ |
|  | DWRUB 52 | DWR17/K551 | 2 | 2007 | Karnal | NWPZ |
|  | DWRB 92 | DWR28/ DWR45 | 2 | 2014 | Karnal | NWPZ |
|  | DWRB 101 | DWR28/BH581 | 2 | 2015 | Karnal | NWPZ |
|  | DWRB 123 | DWRUB54/DWR51 | 2 | 2017 | Karnal | NWPZ |
|  | DWRB 73 | PL710/DWR17 | 2 | 2011 | Karnal | NWPZ |
|  | DWRB 91* | DWR46/ RD2552 | 2 | 2013 | Karnal | NWPZ |
|  | DWRUB 64 | DL472/PL705 | 6 | 2012 | Karnal | NWPZ |
|  | DWRB 137* | DWR28/DWRUB64 | 6 | 2017 | Karnal | NWPZ |
|  | Geetanjali (K 1149) | K12/K572/10// EB410 | 6 | 1997 | Kanpur | NEPZ |
|  | HBL 113 | Selection from Zyphzee | 2 | 1995 | Bajaura | NHZ |
|  | HBL 276 | HBL233/ HBL238 | 6 | 1999 | Bajaura | NHZ |
|  | HBL 316 | Mutant of HBL98 | 6 | 1995 | Bajaura | NHZ |
|  | HBL 391 (Gokul) | HBL316/SONU | 2 | 2010 | Bajaura | NHZ |
|  | Himani | EB489/KAILASH // BHS 15-88 | 6 | 1973 | Shimla | NHZ |
|  | HUB 113 | Karan280/ C138 | 6 | 2014 | Varanasi | NEPZ |
|  | Jagarati (K 287) | KI38/P103 | 6 | 1985 | Kanpur | NEPZ |
|  | JB 58 | RD2615/DL70/ BG105 | 6 | 2005 | Rewa | CZ |
|  | Jawahar Barley 1 (JB 110) | LAKHAN / PL512 | 6 | 2010 | Rewa | CZ |
|  | Jyoti | K12/C251 | 6 | 1974 | Kanpur | NEPZ |
|  | K 141 | K18/IB254 | 6 | 1982 | Kanpur | NEPZ |
|  | K 409 | Jyoti / DL85 | 6 | 2001 | Kanpur | NEPZ |
|  | K 508 (Pragati) | K394/K141 | 6 | 1998 | Kanpur | NEPZ |
|  | K 551 (Ritambhara) | P464/JYOTI | 6 | 1998 | Kanpur | NEPZ |
|  | K 560 (Haritma) | K404/ DL479 | 6 | 1998 | Kanpur | NEPZ |
|  | K 603 | K257/ C138 | 6 | 2001 | Kanpur | NEPZ |
|  | Kailash | EB438/NP100 | 6 | 1975 | Shimla | NHZ |
|  | Karan 16 | AZAM (DWARF)1 / EB7576 | 6 | 1988 | Karnal | NWPZ |
|  | Kedar (DL 36) | BG1/K71 | 6 | 1985 | New Delhi | NEPZ |
|  | Lakhan (K 226) | K12/1B226 | 6 | 1985 | Kanpur | NEPZ |
|  | LSB 2 | Introduction as USA 94 | 6 | 1971 | Palampur | NHZ |
|  | Manjula (K 329) | K4126/SOHAN | 6 | 1997 | Kanpur | NEPZ |
|  | Norboo | Sel from NBL-11 | 6 | 2005 | Leh | NHZ |
|  | NB 1 (NDB 209) | Karan15/P408 | 6 | 2001 | Ayodhya | NEPZ |
|  | NB 2 (NDB 940) | DL470/ RD2035 | 6 | 2001 | Ayodhya | NEPZ |
|  | NB 3 (NDB 1020) | K425/Jyoti | 6 | 2002 | Ayodhya | NEPZ |
|  | NDB 1173 | BYTLRA3 (94-95) /NDB217 | 6 | 2005 | Ayodhya | NEPZ |
|  | NB 5 (NDB 943) | K1178/ Karan748 | 6 | 2009 | Ayodhya | NEPZ |
|  | NDB 1445 | NDB940/Ratna | 6 | 2014 | Ayodhya | NEPZ |
|  | PRB 502 | Selection from local germplasm | 6 | 2010 | Pantnagar | NHZ |
|  | PL 56 | Mutant of C164 | 6 | 1978 | Ludhiana | NWPZ |
|  | PL 172 | RD178/DW 472 | 6 | 1987 | Ludhiana | NWPZ |
|  | PL 419 | PL101/ BH182 | 6 | 1996 | Ludhiana | NWPZ |
|  | PL 426 | Karan92/ PL101 | 6 | 1996 | Ludhiana | NWPZ |
|  | PL 751 | K226/PL226 | 6 | 2007 | Ludhiana | CZ |
|  | PL 807 | 30^th^ IBON-13 (LENT/BLLU// PINON) | 6 | 2012 | Ludhiana | NWPZ |
|  | Raj Kiran (RD 387) | RDB1/ MORROCAINE | 6 | 1982 | Durgapura | NWPZ |
|  | Ranjit (DL 70) | BG1/MEX5-13 | 6 | 1977 | New Delhi | NWPZ |
|  | Ratna | Selection from local material | 6 | 1975 | New Delhi | NEPZ |
|  | RD 31 | RS17/PRIOR | 6 | 1978 | Durgapura | NWPZ |
|  | RD 57 | RS17/PRIOR | 6 | 1978 | Durgapura | NWPZ |
|  | RD 103 | RDB1/K18 | 6 | 1978 | Durgapura | NWPZ |
|  | RD 2035 | RD137/PL101 | 6 | 1994 | Durgapura | NWPZ |
|  | RD 2052 | Api-CM-67/SO -727// PL101 | 6 | 1991 | Durgapura | NWPZ |
|  | RD 2503 | RD103/BH153 // RD2046 | 6 | 1997 | Durgapura | NWPZ |
|  | RD 2508 | RD2035/P409 | 6 | 1997 | Durgapura | NWPZ |
|  | RD 2552 | RD2035/DL472 | 6 | 2000 | Durgapura | NWPZ |
|  | RD 2592 | RD2503/UBL9 | 6 | 2004 | Durgapura | NWPZ |
|  | RD 2624 | Bilara2/ RD2508 | 6 | 2004 | Durgapura | NWPZ |
|  | RD 2660 | RD2052/ RD2566 | 6 | 2006 | Durgapura | NWPZ |
|  | RD 2668 | RD2035/ BCU73 | 2 | 2007 | Durgapura | NWPZ |
|  | RD 2715 | RD387/BH602 // RD2035 | 6 | 2009 | Durgapura | CZ |
|  | RD 2786 | RD2634/NDB 1020// K425 | 6 | 2013 | Durgapura | CZ |
|  | RD 2794 | RD2035/ RD2683 | 6 | 2016 | Durgapura | NWPZ |
|  | RD 2849 | DWRUB52/PL705 | 2 | 2016 | Durgapura | NWPZ |
|  | RD 2899* | -Nil- | 6 | -Nil- | Durgapura | NWPZ |
|  | RD 2907* | -Nil- | 6 | -Nil- | Durgapura | NWPZ |
|  | RDB 1 | Mutant of RS 17 | 6 | 1974 | Durgapura | NWPZ |
|  | RS 6 | RS17/NP21 | 6 | 1978 | Durgapura | CZ |
|  | NBL 11 (Sindhu) | Sel from Sermo Tok Tok | 6 | 2005 | Leh | NHZ |
|  | HBL 87 (Sonu) | Selection from EB233 / GIZA117 | 6 | 1982 | Bajaura | NHZ |
|  | UPB 1008 | HIGO/LINO/3/ CHANICO/ TOCTE// CONGONA/4/... | 2 | 2011 | Pantnagar | NHZ |
|  | Vijaya | K12/C251 | 6 | 1972 | Kanpur | NEPZ |
|  | VLB 1 | NP109/HBL62 | 6 | 1985 | Almora | NHZ |
|  | VLB 56 | Morocco/VLB1 | 6 | 2005 | Almora | NHZ |
|  | VLB 85 | HBL348/VLB49 | 6 | 2007 | Almora | NHZ |
|  | VLB 94 | DL237/VLB58 | 6 | 2016 | Almora | NHZ |
|  | VLB 118 | 14^th^ EMBSN -9313 | 6 | 2014 | Almora | NHZ |
|  | DWRB 160 (Karan Maltsona)* | -Nil- | 2 | -Nil- | Karnal | NWPZ |
|  | HBL 713 (Him Palam Jau 1)* | -Nil- | 6 | -Nil- | Bajaura | NHZ |
|  | VLB 130* | -Nil- | 2 | -Nil- | Almora | NHZ |
|  | DWRB 182* | -Nil- | 2 | -Nil- | Karnal | NWPZ |
|  | PL 891* | -Nil- | 2 | -Nil- | Ludhiana | NWPZ |
|  | KB 1425** | -Nil- | -Nil- | -Nil- | -Nil- | Unknown |
|  | BK 303** | -Nil- | -Nil- | -Nil- | -Nil- | Unknown |
|  | BK 306** | -Nil- | -Nil- | -Nil- | -Nil- | Unknown |
|  | BK 316** | -Nil- | -Nil- | -Nil- | -Nil- | Unknown |
|  | BCU 2241** | -Nil- | -Nil- | -Nil- | -Nil- | Unknown |

NWPZ: North Western Plain Zone of India; NEPZ: North Eastern Plain Zone of India; NHZ: Northern Hill Zone of India: CZ: Central Zone of India; *Geographical region estimated based on the location of centre by which the genotype was developed; **Data unavailable (Unknown)

***Table S2:*** *Grading system followed for barley genotypes screening against* R. maidis

| **Grading based on aphid population (Scale 1 to 5)** | | |
| --- | --- | --- |
| **Grade/Scale** | **Number of aphids per shoot** | **Rating/Reaction** |
| 1 | 0 | Immune (I) |
| 2 | 1 to 5 | Resistant (R) |
| 3 | 6 to 10 | Moderately Resistant (MR) |
| 4 | 11 to 20 | Susceptible (S) |
| 5 | 21 and above | Highly Susceptible (HS) |
| **Grading based on leaf chlorosis (Scale 1 to 5)** | | |
| **Grade/Scale** | **Leaf chlorosis** | **Rating/Reaction** |
| 1 | No chlorosis | Immune (I) |
| 2 | Less than 1/3 of leaf area chlorotic | Resistant (R) |
| 3 | 1/3 to 2/3 leaf area chlorotic | Moderately Resistant (MR) |
| 4 | More than 2/3 of leaf area chlorotic | Susceptible (S) |
| 5 | Necrosis in at least one full leaf | Highly Susceptible (HS) |
| **Grading based on leaf rolling (Scale 1 to 3)** | | |
| **Grade/Scale** | **Leaf rolling** | **Rating/Reaction** |
| 1 | No rolling | Resistant (R) |
| 2 | Trapping or curling in one or more leaves | Moderately Resistant (MR) |
| 3 | Rolling in one or more leaves | Susceptible (S) |

**Table S3:** Characterization of barley genotypes into various categories of plant resistance based on AII.

| **Grade** | **Reaction** | **No. of Genotypes** | **Genotypes** |
| --- | --- | --- | --- |
| **1** | **I** | **0** | -Nil- |
| **2** | **R** | **2** | RD 2849 and DWRUB 52 |
| **3** | **MR** | **20** | Himani, K 560 (Haritma), PL 807, Raj Kiran (RD 387), Ranjit (DL 70), Ratna, RD 31, RD 57, RD 103, RD 2035, RD 2052, RD 2899, HBL 87 (Sonu), UPB 1008, VLB 1, VLB 85, VLB 94, HBL 713 (Him Palam Jau 1), BK 303 and BK 316 |
| **4** | **S** | **58** | Amber (K 71), BCU 73 (Rekha), BH 75, BH 393, BH 885, BH 902, BH 946, BH 959, BHS 169, BHS 400, Bilara 2, Clipper, DL 88, Dolma, DWR 28, DWRB 101, DWRB 91, DWRUB 64, DWRB 137, Gitanjali (K 1149), HBL 113, HUB 113, Jagarati (K 287), JB 58, JB 1, K 141, K 508 (Pragati), K 551 (Ritambhara), K 603, Kailash, Karan 16, Kedar (DL 36), Lakhan (K 226), LSB 2, NB 1 (NDB 209), NB 2 (NDB 940), NB 3 (NDB 1020), NDB 1173, PRB 502, PL 172, PL 419, PL 426, PL 751, RD 2503, RD 2508, RD 2786, RD 2794, RDB 1, RS 6, NB 11 (Sindhu), Vijaya, VLB 118, DWRB 160 (Karan Maltsona), VLB 130, DWRB 182, PL 891, BK 306 and BCU 2241 |
| **5** | **HS** | **29** | Alfa 93, Azad (K 125), BG 25, BG 105, BHS 46, BHS 352, BHS 380, DWRB 92, DWRB 123, DWRB 73, HBL 276, HBL 316, HBL 391 (Gokul), Jyoti, K 409, Manjula (K 329), Norboo, NB 5 (NDB 943), NDB 1445, PL 56, RD 2552, RD 2592, RD 2624, RD 2660, RD 2668, RD 2715, RD 2907, VLB 56 and KB 1425 |
